# Inter-examiner reliability of radiographic measurements from Open-mouth lateral bending cervical radiographs

**DOI:** 10.1186/s12998-020-00317-6

**Published:** 2020-05-26

**Authors:** Karthik V. Hariharan, Lauren Terhorst, Matthew D. Maxwell, Christopher G. Bise, Michael G. Timko, Michael J. Schneider

**Affiliations:** 1 Department of Physical Therapy, University of Pittsburgh, 6046 A Forbes Tower, Pittsburgh, PA 15260 USA; 2 Department of Occupational Therapy, University of Pittsburgh, Pittsburgh, PA USA; 3 Interventional Spine and Sports Medicine, MedStar National Rehabilitation Network, School of Medicine, Georgetown University, Washington, DC, USA; 4 Division of Physical Therapy, West Virginia University, Morgantown, WV USA

**Keywords:** Cervical spine injury, Craniocervical junction, Radiography, Instability, Hypermobility

## Abstract

**Background:**

Following head and neck trauma, the involvement of the cranio-cervical junction (CCJ) and its contribution to a patients transition to chronic pain, is poorly understood. The detection of hypermobility in this region is dependent on clinical examination and static imaging modalities such as x-ray, CT and MRI. Sagittal plane hypermobility of the CCJ is evaluated using saggital view, flexion-extension cervical radiographs. Frontal plane hypermobility is typically assessed using lateral bending and open mouth cervical radiographs. Unfortunately there is no established reliability surrounding the use of these measures. This study explores the reliability of radiographic measurements of lateral-bending open-mouth cervical radiographs.

**Methods:**

Cervical open-mouth lateral-bending X-ray images were collected from 56 different patients between 18 and 60 years of age patients following cervical spine injury. These images were interpreted by two musculoskeletal radiologists and two clinicians (physiatrist and chiropractor), using a standard set of measurements. Measurements included qualitative and quantitative assessments of the amount of asymmetry noted between various osseous landmarks. Reliability statistics were calculated for levels of agreement using kappa coefficients (κ) and Intraclass Correlation Coefficients (ICC) for dichotomous and continuous variables, respectively.

**Results:**

Reliability (κ) for qualitative assessments were moderate to substantial for asymmetry of neutral C2 spinous position, dens-lateral mass spacing, and “step off” between the lateral borders of the articular pillars of C2 and C1 lateral mass (κ range = .47–.78). ICC values for the quantitative measurements of dens-lateral mass spacing and “step off” between the lateral borders of the C2 articular pillars and C1 lateral mass were moderate to excellent (ICC range = .56–.97).

**Conclusions:**

The qualitative and quantitative measurements used in this study demonstrated good to excellent inter-examiner reliability. Correlation with clinical findings is necessary to establish the utility of these measurements in clinical practice.

## Background

Cervical spine injury complicates the care of approximately 4% of patients admitted to trauma centers across the United States [1]. Early diagnosis of these injuries is imperative as delayed or missed diagnoses result in increased morbidity and mortality [2]. Given the limited utility of standard radiographs, more extensive radiographic studies are often performed including supine oblique views, flexion-extension radiographs, and computed tomography (CT) [3]. CT used routinely in trauma patients has improved recognition of cervical fractures but ligamentous injuries can still be missed, as they cannot be easily visualized on CT [4]. CT or magnetic resonance imaging (MRI) may demonstrate articular subluxation or directly visualize ligamentous injury [5–7]. There are concerns however, surrounding technical considerations and the reliability of these tools [8–13]. Given that injury to the cranio-cervical junction (CCJ) may lead to chronic symptoms following trauma, the role of CCJ injury is gaining greater recognition in non-responders to initial management of common injuries such as concussion and whiplash [5, 6, 8, 9, 14].

It is important to note the important distinction between the findings of CCJ ‘clinical instability’ versus ‘hypermobility’, which arguably represent different stages along a stability continuum. Clinical instability, as described by Panjabi and White, is the “loss of the ability of the spine under physiologic loads to maintain its pattern of displacement so that there is no initial or additional neurological deficit, no other major deformity, and no incapacitating pain.” [15] Cervical spine ‘hypermobility’ is increased segmental motion ostensibly due to a sprain of the cervical ligaments, where the injury does not cause clinical instability but may cause persistent symptoms of neck pain and cervicogenic headache.

The use of lateral views of the upper cervical spine with flexion and extension views was first described by Hohl and Baker in 1964 [16] and is now used to assess atlantoaxial hypermobility [17, 18]. However, these views are limited to detecting hypermobility in the sagittal plane. The use of lateral flexion-extension stress radiographs to measure the atlantodental interval (ADI) in the neutral and full-flexion / extension positions is considered a gold standard diagnostic test for determining CCJ hypermobility in the sagittal plane. Wellborn et al. suggested that any ADI greater than 3 mm is concerning [19]. However, there are no standardized normative values for measurements of the lateral displacements of C1 in the frontal plane when measured from anteroposterior open-mouth (AP-OM) radiographs obtained at end range lateral bending [20]. One study using dynamic AP-OM lateral bending radiographs reported atlanto-dental lateral shift as an indicator of C1–2 hypermobility in patients with rheumatoid arthritis [21]. They reported that dynamic hypermobility of the C1–2 joint seen on lateral bending radiographs, had the same prevalence as anterior C1–2 hypermobility seen on flexion radiographs. The authors concluded that excessive movement in either the sagittal plane or frontal plane was useful for making the diagnosis of early atlanto-axial disease in that population. The use of anterior-posterior open-mouth (AP-OM) lateral bending views may prove to be a complementary cost-effective technique for evaluating excessive movement of the CCJ in the frontal plane; however, it has not been validated in patients with suspected post-traumatic hypermobility.

While lateral cervical flexion-extension radiography is considered reliable and valid in detecting CCJ hypermobility in the sagittal plane, no reliability data exist in evaluating CCJ hypermobility in the frontal plane. Reliability of procedures evaluating frontal plane stability measures must first be established, prior to conducting population studies comparing normative data with injured populations. This study will provide baseline data for the purpose of establishing the interrater reliability of measurements of open mouth atlantoaxial radiographs with dynamic lateral bending views.

## Methods

The study methods were approved by the University of Pittsburgh IRB (PRO13040355) prior to data collection. The study was given ‘exempt’ status as it was based upon a retrospective review of existing medical records. Cervical open-mouth lateral-bending X-ray images from 56 patients were collected retrospectively by a Picture Archiving and Communication System (PACS) query, cross-referencing physicians evaluating the designated population and cervical radiograph orders related to the study of interest. This sample size was based on power analysis (80%; alpha = 0.05) to detect a Kappa and I.C.C for dichotomous and continuous variables respectively. The patient population included patients who had been referred for a series of cervical radiographs, including open-mouth lateral bending stress views for evaluation of persistent symptoms related to head and/or neck trauma.

**Fig. 1:**
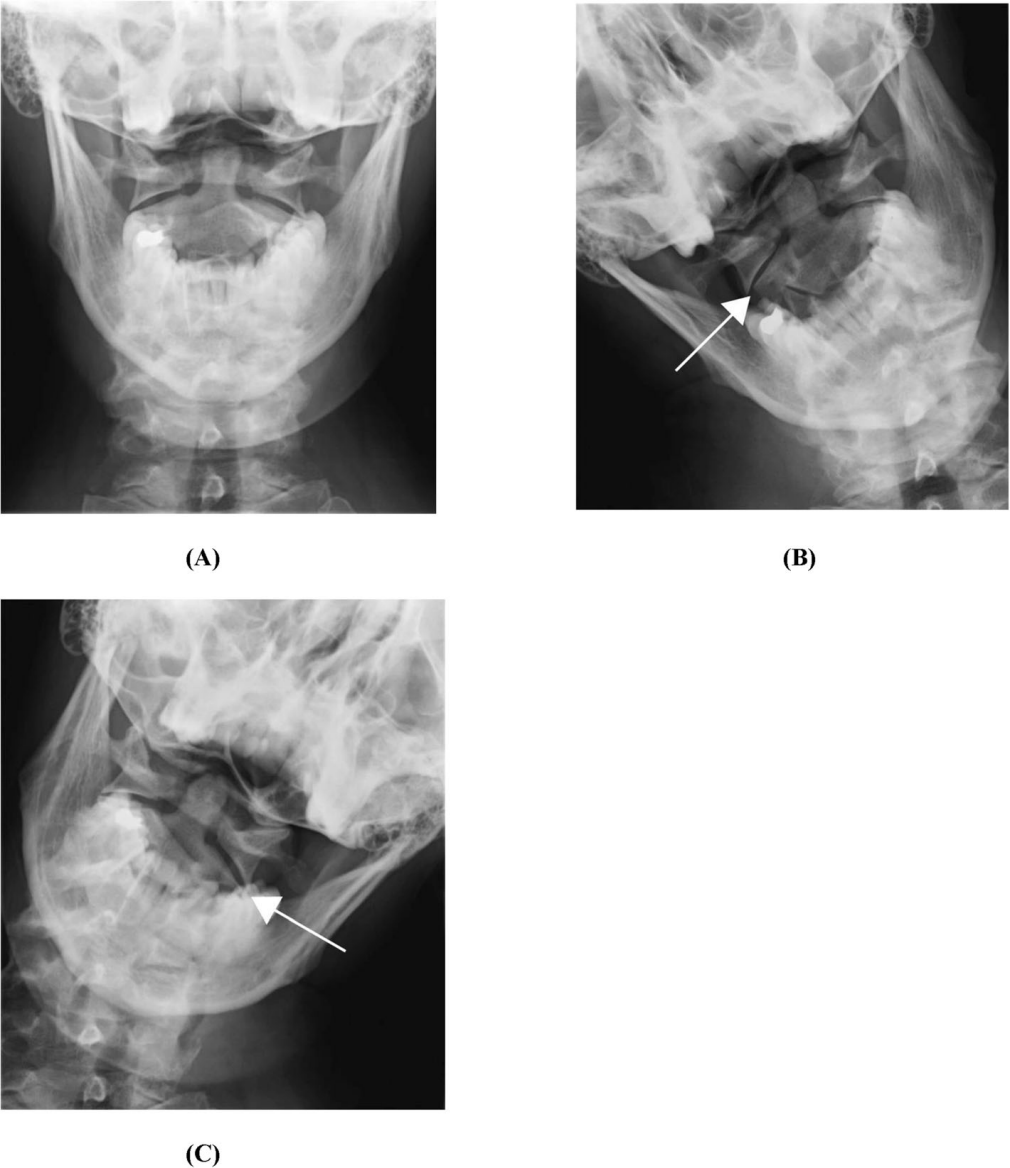
Open mouth Anteroposterior Lateral bending cervical spine radiographs in neutral (**a**); right side bending (**b**); left side bending (**c**) [20]

The images were then de-identified and screened by one author to ensure that only appropriate studies were included. Images included were from patients between the ages of 18–60 years who had experienced head and/or neck injury, and had persistent symptoms necessitating evaluation by a physician. Patients were excluded if radiographic evidence of prior cervical surgery was noted during preliminary image review. Collected radiographic images included: anteroposterior open mouth (AP-OM) view, AP-OM views with lateral bending view to both sides, as well as AP and lateral views of the entire cervical spine. Figure 1a, b and c are images obtained from the study conducted by Mathers et al. [20]. A similar set of Cervical Radiographic images obtained from the PACS system. In some cases, flexion-extension lateral views of the cervical spine were included in the radiographic series, however they were not assessed during the data review for the purposes of this study.

After de-identification, images were assigned a random number. De-identified images were then interpreted by two musculoskeletal radiologists (raters 1 & 2) and two clinicians (physiatrist and chiropractor, raters 3 & 4). The study was originally designed to include data interpretation only by the two radiologists scheduled to interpret the radiographic images but we later decided to also include data interpreted by two clinicians to increase the generalizability of the results. All four raters had varying years of experience (6 to 24 years) in their respective areas of specialty (Radiology; Physical Medicine and Rehabilitation; and Chiropractic Medicine). There was no plan to perform any specific between-provider comparisons. By nature of study design, interpreters were not blinded to the history of head and/or neck trauma. Interpreters were blinded to all other clinical information used to include study subjects, as well as to any previous measurements taken in clinical assessment.

**Fig. 2:**
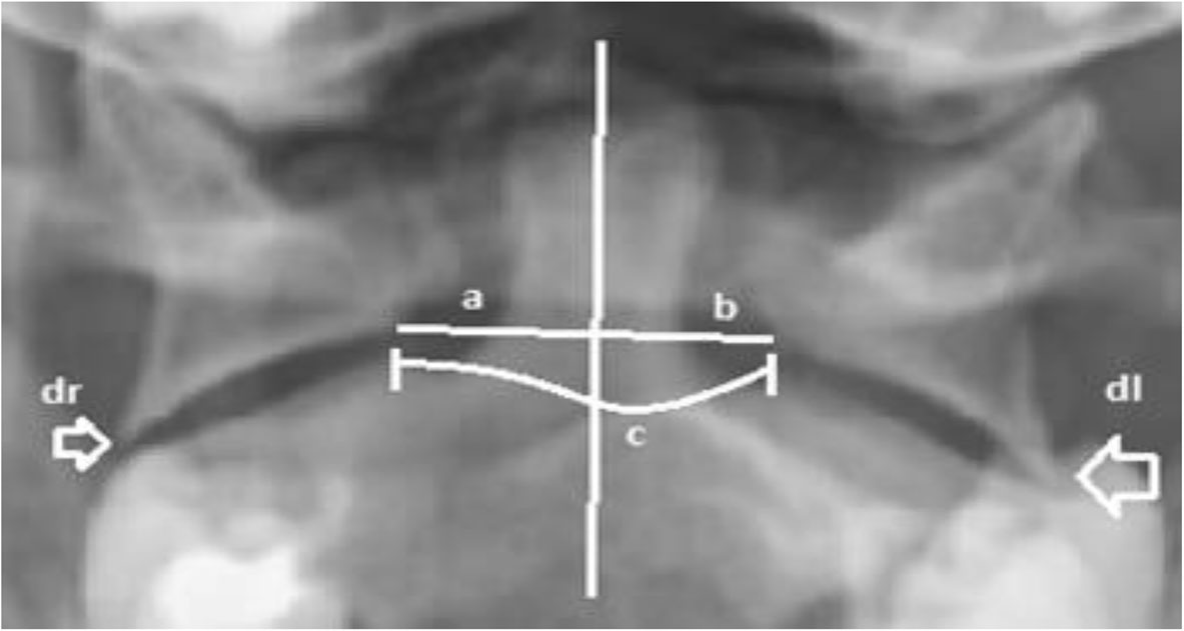
Open Mouth Lateral bending cervical spine radiographs with measures recorded. **a** midline of Dens to right lateral mass; **b** midline of Dens to left lateral mass; **c** width between lateral mass; dr: Right lateral mass step-off; dl: left lateral mass step-off

**Fig. 3:**
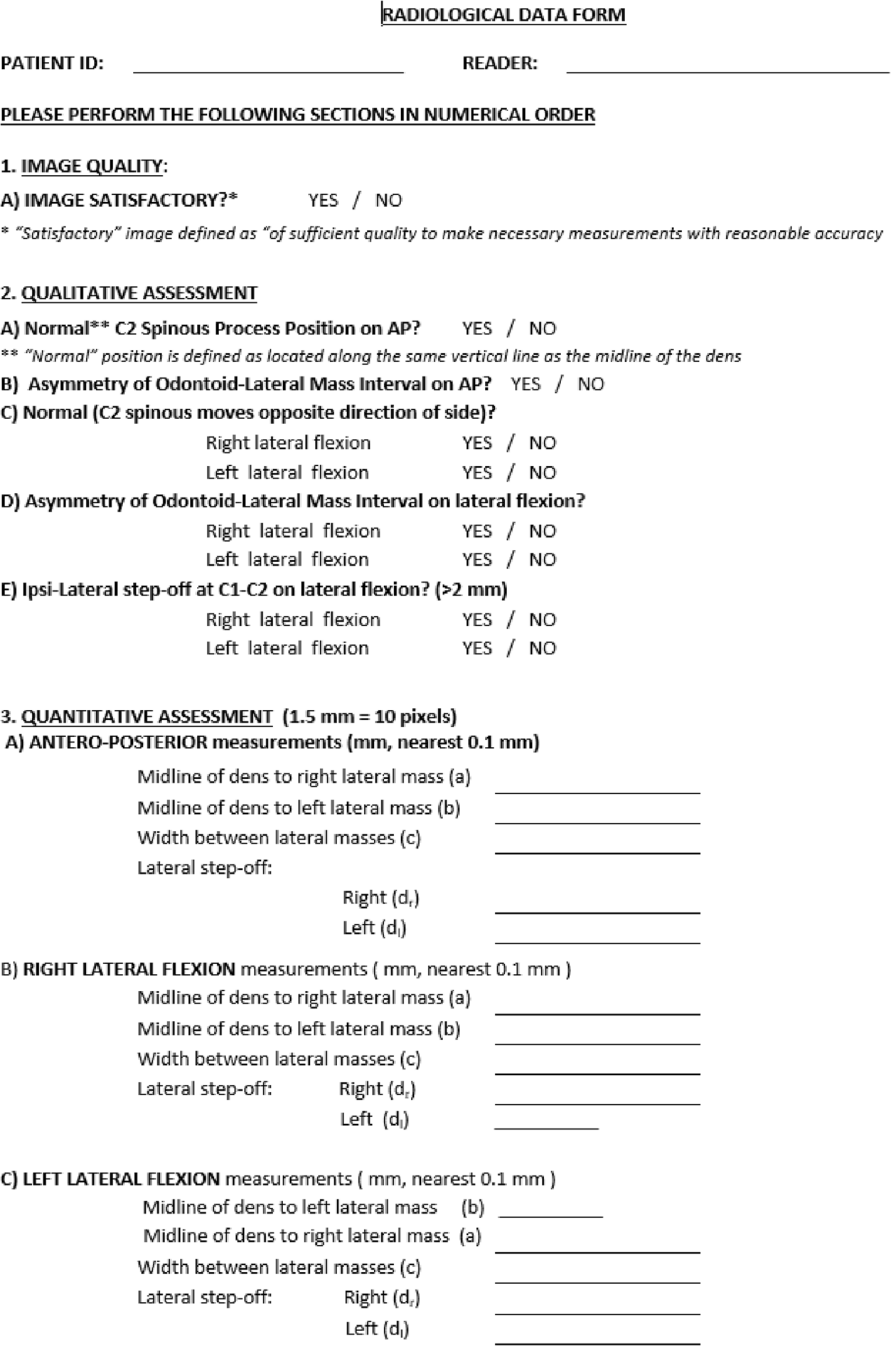
Radiological data form

Prior to study initiation, the authors completed an extensive literature review to identify appropriate quantitative and qualitative measurement techniques for hypermobility of the C1-C2 articulation. These techniques were then given to the assessors for review and discussion. As a group, the assessors evaluated the strength of the evidence and applied their clinical experience to arrive at a consensus for measurement inclusion. These measurements included qualitative and quantitative assessments of the degree of asymmetry noted between various osseous landmarks on the AP-OM views (Fig. 2). The qualitative assessments were made by visual observation or the ‘eye-ball method’, which included a series of dichotomous questions regarding: the quality of the images (good/poor); C2 spinous process movement (normal/abnormal); asymmetry of the para-odontoid (symmetrical/asymmetrical); and lateral step-off of the lateral mass of C1 on the body of C2 (present/absent). Quantitative assessments involved measurements (in millimeters) of the spacing between the medial edge of the C1 lateral masses and midline of the dens, as well as the amount of step-off (in millimeters) between the lateral edge of the lateral masses and the body of C2 (Fig. 2). These findings were recorded on the Radiology Data Form This form was provided to all assessors (Fig. 3) and outlined the assessment method and data collection for each measurement (Fig. 3).

Each examiner used a dedicated PACS workstation equipped with high definition monitors; each clinician used a standard computer monitor with self-selected settings in normal ambient light, as would be typical in a clinical setting. Quantitative measures were assessed using a Picture Archiving and Communicating System-Digital Imaging and Communications in Medicine (PACS-DICOM) viewing software (RadiAnt). The specific tool within the software, ‘Segment Length’, allows a reader to measure distance or intervals between two points, drawn electronically by the viewer. The software provides distances measured in pixels and millimeters, the latter of which was used for standardization. Radiographic image measurements were then compiled by an honest broker for statistical analysis to ensure blinding of all individuals involved in image interpretation for this study.

Data were analyzed using Statistical Packages for the Social Sciences (SPSS, v.23) to determine levels of inter-observer agreement. Reliability was calculated using the ICC for consistency (two-way mixed, single measure model: ICC 3.1) for radiographic data expressed as continuous variables. Consistent deviations in measures between observations or observers (bias) were examined by inspecting the 95% confidence intervals of the differences between measurements. For categorical data, reproducibility was calculated using the kappa coefficient. Kappa can be influenced by the case distribution (attribute prevalence) and bias. Therefore, the kappa coefficients were adjusted for prevalence and bias [5, 16]. Agreement was determined by calculating the standard error of measurement. (κ) coefficients of .41–.60 were considered moderate, values of .61–.80 were considered substantial, and values greater than .80 were considered excellent [22]. ICC values of 0.5 were considered moderate, while values greater than 0.8 were considered strong or excellent [23].

## Results

Table 1 demonstrates that the reliability for the qualitative assessments were moderate to substantial (κ range = .42–.70) for asymmetry of neutral C2 spinous position and dens-lateral mass spacing, as well as for “step off” between the lateral borders of the C2 articular pillars and C1 lateral mass (κ range = .47–.78). One unusual finding was the extremely high prevalence of normal spinous movement, which confounded calculation of kappa statistics for this particular measurement. In our 56 cases, the vast majority of images showed normal C2 spinous movement to opposite side of lateral bending, which may have artificially inflated the raw percentage of agreement, thereby confounding calculation of the kappa statistic due to the high prevalence of this finding.

**Table 1:** Summary of all kappa statistics for all dichotomous variables

Item	Raters 1 & 2		Raters 3 & 4	
	κ	% Agree	κ	% Agree
Image Satisfactory	.278	64.8	.393	68.6
Spinous Position	.641	84.3	.537	84.3
Asymmetry of Odontoid-Lateral Mass	.424	74.4	.698	91.3
Normal spinous movement -right	^a^N/C	81.8	^a^N/C	97.9
Normal spinous movement-left	^a^N/C	97.1	^a^N/C	97.9
Midline of dens to right lateral mass	.393	67.5	.424	72.5
Midline of dens to left lateral mass	.302	62.2	.697	85.7
step off right	.566	78.6	.465	75.6
step off left	.778	89.2	.665	84.4

^a^N/C Not Calculated. The extremely high prevalence of normal spinous movement during lateral bending confounded the calculation of kappa statistics for this measurement

Table 2 demonstrates that data for the quantitative measurements of atlantoaxial movement were more robust, showing moderate to excellent reliability (ICC range = .56–.97).

**Table 2:** Summary of all ICC statistics for all continuous variables

M	Raters 1, 2	95% CI	Raters 3, 4	95% CI
AP Midline of dens to right lateral mass	.913	(.841, .952)	.925	(.864, .958)
AP Midline of dens to left lateral mass	.935	(.883, .965)	.927	(.869, .960)
AP Width	.929	(.871, .961)	.867	(.760, .926)
AP step off right	.581	(.238, .770)	.892	(.805, .940)
AP step off left	.768	(.574, .873)	.880	(.784, .934)
RLF Midline of dens to the right lateral mass	.922	(.854, .958)	.949	(.904, .973)
RLF Midline of dens to the left lateral mass	.934	(.878, .964)	.928	(.867, .961)
RLF Width	.943	(.894, .969)	.954	(.914, .976)
RLF step off right	.882	(.784, .936)	.941	(.893, .967)
RLF step off left	.748	(.532, .865)	.799	(.632, .890)
LLF Midline dens to the left lateral mass	.830	(.689, .907)	.854	(.731, .921)
LLF Midline dens to the right lateral mass	.859	(.741, .923)	.785	(.611, .881)
LLF Width	.908	(.830, .950)	.921	(.854, .957)
LLF step off right	.556	(.153, .767)	.792	(.625, .885)
LLF step off left	.884	(.785, .938)	.968	(.942, .982)

## Discussion

The clinical utility of imaging techniques to evaluate suspected traumatic injury to the cranio-cervical junction (CCJ) continues to be debated in recent literature. Findings from this study add to the body of literature examining the use of dynamic radiographs to evaluate segmental stability of the cervical spine.

To our knowledge, the inter-rater reliability of AP-OM lateral bending radiograph interpretation has not been previously assessed in any population. The technique requires precise patient positioning to ensure accuracy and minimize the degree to which overlapping structures interfere with interpretation. These technical considerations introduce potential variability in interpreting these images, their associated measurements, and their clinical significance. AP-OM dynamic lateral bending radiographs may represent an additional tool in evaluating atlantoaxial hypermobility, visualizing increased atlantoaxial movement under end-range stress conditions [14, 20, 21] Some studies have proposed that such radiographic findings may be associated with pathology, one of which was performed in a population of patients with refractory symptoms after mild traumatic brain injury [14]. Increased motion may be visualized if alar ligament laxity is present – seen as an ipsilateral “offset” adjacent to the lateral C1-C2 joint and corresponding inset of the contralateral side with minimal C2 spinous rotation. Additionally, a step off of greater than 1–2 mm at the lateral margin of the lateral C1-C2 joint during ipsilateral bending has been proposed as an indicator of significant hypermobility of the CCJ, although the validity of this measurement has not been investigated.

One previous study examined the use AP-OM lateral bending radiographs to characterize CCJ hypermobility in a cohort of patients diagnosed with rheumatoid arthritis [21]. They used measurements to calculate the amount of atlanto-dental lateral shift (ADLS), ostensibly indicating lateral hypermobility of the C1–2 joint (RA); the data within the RA cohort was compared with healthy controls. They reported that excessive motion of the C1–2 joint on lateral bending radiographs – quantified by the magnitude of ADLS – was just as prevalent as anterior C1–2 hypermobility noted on forward flexion radiographs. The authors concluded the ADLS measurement was sensitive to early atlantoaxial disease in patients who did not yet demonstrate findings in traditional lateral views with flexion, suggesting that AP-OM radiographs are more sensitive to a lesser amount of segmental motion. One follow-up study described a variant on this technique, evaluating ADLS using close-mouth lateral bending views [24]. The technique, while potentially useful, has not yet been evaluated comparing normative data with diseased populations. These findings suggest that AP-OM dynamic views may provide a useful imaging technique, which complements the use of CT and MRI in evaluation of CCJ injury.

One seemingly paradoxical finding was the low Kappa values for normal spinous movement compared with the high raw percentage of overall agreement; this is particularly apparent in the complete inability to compute a kappa value for normal spinous movement left. This paradox relates to the fact that calculation of the kappa statistic is highly sensitive to the prevalence of the observed clinical finding. In our 56 cases, the vast majority of images showed normal C2 spinous movement to opposite side of lateral bending, which may artificially inflate the raw percentage of agreement and confound calculation of the kappa statistic due to the high prevalence of this finding.

There are conflicting reports on the utility of the odontoid lateral mass interspace (OLMI) in predicting occult injury, as measured both on cross sectional imaging and radiographs. It has been shown that static CT imaging often reveals OLMI asymmetry in asymptomatic patients [25]. One recent study found that OLMI as measured on static CT imaging lacks sensitivity and specificity in detecting ligamentous injury, unless optimal technique is employed [26]. When measured on odontoid open-mouth radiographs, OLMI asymmetry may be an indicator of serious occult injury such as rotary subluxation or fracture [27]. Several studies have suggested, however, that OLMI may demonstrate asymmetry of 2 mm or greater in the absence of any pathology [25, 28, 29].

The clinical utility of MRI in evaluating CCJ is questionable and somewhat controversial. Isolated case studies have revealed mixed results in evaluating abnormal signal intensity within the alar and transverse ligaments on fast spin-echo (FSE) T2 and proton density (PD) sequences [6, 8, 9, 30]. Follow-up studies evaluating MRI in Whiplash Associated Disorders (WAD) and other clinically relevant but non-traumatic diagnoses such as chronic neck pain, cervicogenic headache, and migraine headaches have not confirmed any significant correlation with trauma, either acutely or remotely [10, 31, 32]. The majority of these studies evaluated ligamentous injury using a four-point grading scale.

Many authors have noted concerns regarding the use of MRI in evaluating occult CCJ injury. Given the above noted small study sizes, a more recent meta-analysis attempted to evaluate these data using stronger statistical methods [12]. The authors concluded that abnormal imaging findings did not correlate with symptoms and that there was no evidence to support the assertion that MRI reveals ligamentous injury in this patient population. Several studies have revealed issues with the reliability of image interpretation, as well as technical difficulties with patient positioning and image acquisition when performing these sequences [8, 9, 11–13, 33]. Lastly, this imaging technique may be inaccurate unless optimal technique is employed, necessitating expertise by involved staff [11, 13, 26]. These conflicting data highlight the need to investigate complementary imaging techniques which may reveal occult CCJ injury; additional methods of dynamic radiography, as described in this study, may provide that complement. The need for effective and reliable evaluative techniques is underscored by the growing recognition of upper cervical spine injury as a potential underlying factor in the persistent symptoms after concussion injury [14]. Many athletes who sustain a concussion often injure both their neck and head. Among cervical spine injuries, the upper cervical spine has the greatest anatomic connection and neurologic cross-innervation with head [20, 34]. It follows that, in these patients who suffer prolonged headache and dizziness, occult injury of the CCJ complex may be more common than currently suspected and may frequently go unrecognized. It is also important to note that occult traumatic injury to the CCJ has been shown to be more frequent in the pediatric population [35, 36] where concussions are more frequent and symptoms are persistent. Thus, it is imperative that we continue to develop imaging techniques for the cervical spine which complement currently available technology, minimize radiation, and constrain costs when appropriate.

## Limitations

There are several limitations to this study. While the reliability of this imaging measurement technique appears to be good, this study does not provide evidence supporting the validity of the technique in evaluating CCJ injury. The technique involved in obtaining AP-OM radiographs is inherently sensitive to variability between patients and providers; lateral bending views represent a 2D projection of any combination of 3D movements in the coronal, axial, and sagittal planes. In particular, variations in the degree of concurrent atlanto-axial rotation during lateral bending may affect the clinical significance of the measurements. A prospective study comparing this imaging technique with physical examination findings, cross-sectional imaging, and potentially intraoperative evaluation is needed to better characterize the construct validity of this technique.

Additionally, this study did not examine the role that technical considerations play in image acquisition, which has the potential to affect image measurement reliability in clinical practice. For instance, more consistent images might be obtained with the use of a modified cervico-thoracic orthosis which allows only coronal motion during imaging acquisition. We found in the vast majority of cases that normal C2 spinous rotation occurred to the contra-lateral side of lateral bending. In retrospect, we should have asked our evaluators to make a qualitative assessment about presence of symmetrical or asymmetrical C2 movement, rather than asking whether the C2 motion was normal or abnormal. These are important considerations to address as this imaging technique is to be adopted in guiding evaluation and treatment.

## Conclusion

Given the potential role of occult cervical spine injury in prolonged recovery from traumatic injuries to the head and neck, it is important to establish evaluation tools that are reliable, affordable, and provide value as an evaluation tool for occult injury. The use of AP-OM radiographs with lateral bending views may prove to be such a test. This study demonstrated good to excellent interrater reliability of both qualitative and quantitative measurements obtained using this imaging technique. Its use as a valid instrument in the clinical assessment of CCJ injury remains to be established. It does, however, offer potential promise as a relatively inexpensive and minimally invasive screening test for CCJ injury.

## Data Availability

The datasets used and/or analysed during the current study are available from the corresponding author on reasonable request.

## Abbreviations

CCJ: Cranio-cervical Junction
CT: Computer Tomography
MRI: Magnetic Resonance Imaging
ICC: Intraclass Correlation Coefficient
K / κ: Kappa Coefficient
ADI: Atlanto-Dental Interval
AP: Antero-Posterior
AP-OM: Antero-Posterior Open Mouth
IRB: Institutional Review Board
PACS: Picture Archiving & Communication System
DICOM: Digital Imaging and Communications in Medicine.
ADLS: Atlanto-Dental Lateral Shift
OLMI: Odontoid Lateral Mass Interspace
PD: Proton Density
WAD: Whiplash Associated Disorders
RLF: Right Lateral Flexion
LLF: Left Lateral Flexion
FSE: Fast Spin Echo
